# Data analysis about Diabetes Mellitus and glycemic control collected from screening and prevention campaigns of Chronic Kidney Disease in a community-based service and education

**DOI:** 10.1186/1758-5996-7-S1-A198

**Published:** 2015-11-11

**Authors:** Lucas Arnaud, João Amarildo Rodrigues da Silva Filho, Mateus Henrique Mendes, Carina Vieira de Oliveira, Karísia Santos Guedes, Levi Oliveira de Albuquerque, Caio César Jucá Lucena, Laio Lopes Ladislau Lima, Jayne Maria Bernardo dos Santos, Leandro Costa Lima, Maurocelio Rocha Pontes Filho, Mateus Albuquerque Azevedo, Carolina Teles de Macedo, Isadora Rodrigues da Costa, Thaís Amanda Silva Pereira Castelo Branco, Elizabeth de Francesco Daher

**Affiliations:** 1 UFC, Fortaleza, Brazil

## Background

Chronic Kidney Disease (CKD) has been prominent in the global health scene as important factor of morbidity and mortality among the population, moving large amounts of investiments and burdening the health care system with their treatment. In that context, Diabetes Mellitus is a major risk factor, reason why the campaigns also collected data related to that subject. SETTINGS: The LPDR group acts in the prevention of CKD by conducting educational and screening campaigns in different communities of the state.

## Objectives

Identify the prevalence of Diabetes Mellitus and CKD, its risk factors and comorbidities in populations of cities in the state of Ceará, diffusing prevention of kidney disease through better understanding of this disease and promoting exchange of experiences between students and community.

## Materials and methods

Screening tests for risk factors for DM and CKD were made in more than 7 cities and people were informed about those diseases and how to avoid it. Individuals whose tests showed risk factors were referred for clinical follow-up in health facilities nearby, after being informed about the importance of monitoring their comorbidities.

## Results

In the 5 yrs. of work, 3187 people were seen in more than 7 cities( 52,8% of them being female). In this population, 33 (1.03%) of individuals who claimed to be healthy were actually possible carriers of Diabetes Mellitus. The data showed that 397(12,45%) had diagnosed DM, and 1534 (48,13%) had family history of DM. 256(8.03%) had both DM and family history for DM. The data shows that DM is underdiagnosed and poorly controlled in many patients, and that the correct use of anti-diabetic medication is not strictly followed by a significant number of individuals. 213 had diagnosed DM, but had dangerous hyperglycemia (>140) (measured by CBG), and 221 didn't had diagnosed DM, but had dangerous hyperglycemia. 109 had diagnosed DM and heavily dangerous hyperglycemia (>200). 166 had diagnosed DM and claimed to regularly practice exercises, and 228 had DM but don't do exercises.

## Conclusions

This data analysis shows the urgent need of population education about DM, glycemic control and anti-diabetic medication. It shows that many people claim to be healthy, but are possible carriers of DM or have dangerous or heavily dangerous hyperglycemia. It also shows that many people with diagnosed DM doesn't know about the correct use of anti-diabetic medication.

